# Domestication modifies the volatile emissions produced by male Queensland fruit flies during sexual advertisement

**DOI:** 10.1038/s41598-018-34569-3

**Published:** 2018-11-07

**Authors:** Jeanneth Pérez, Soo Jean Park, Phillip W. Taylor

**Affiliations:** 1 Department of Biological Sciences, Macquarie University, Sydney, NSW 2109 Australia; 2 Department of Molecular Sciences, Macquarie University, Sydney, NSW 2109 Australia; 3 Australian Research Council Industrial Transformation Training Centre for Fruit Fly Biosecurity Innovation, Macquarie University, Sydney, NSW 2109 Australia

## Abstract

Insects commonly undergo substantial changes during adaptation for laboratory or mass-rearing environments (‘domestication’) that may have significant implications for inferences from laboratory studies and utility for biological control. We assessed the effect of domestication on the amount and blend of volatiles released during sexual calling by laboratory-reared *Bactrocera tryoni* males using colonies from three regions of Australia: Brisbane, Cairns and Sydney. For each region, volatiles released by males from a young colony (five or fewer generations) and an old colony (20+ generations) during sexual calling was compared. Males from old colonies released more volatiles than males from young colonies. All components of the blend were more abundant in one or more of the older colonies, although differences varied by compound and by region. To assess changes over generations, the young and old colonies obtained from Brisbane were sampled at 5, 12 and 15 generations (young colony) and 25, 35 and 38 generations (old colony). While the old colony remained unchanged, flies from the young colony released more volatiles at each sequential sampling episode, and became increasingly similar to the old colony. Increased volatile production during domestication may be an adaptive response to crowded rearing conditions in which males need to overcome a chemically noisy environment to be sexually successful.

## Introduction

Insects are commonly maintained for many generations under artificial rearing conditions in laboratories for research or in factories to generate large numbers for biological control. Artificial rearing conditions represent a new environment that is vastly different from nature, with very different selection pressures, such that over generations the reared insects tend to diverge adaptively from their wild origins (‘domestication’)^1–3^. Such evolutionary changes can constrain the inferences about nature that can be reliably drawn from studies of domesticated insects^4,5^ and can also constrain the ability of insects to thrive and mate if released back into the field^6–8^.

Domestication of tephritid fruit flies for laboratory or factory settings has been found to have significant effects on major life history traits, environmental tolerance, and sexual performance. Domesticated fruit flies tend to have faster development, maturing at much younger ages than wild type flies^9–14^, increased fecundity^4,15^, decreased lifespan^4,16^, modified diurnal patterns of sexual activity^17^, reduced ability to evade predators^18^, and reduced sexual competitiveness^19–21^. Sexual advertisement and mating are complex in fruit flies, with males of most species using repertories of visual, acoustic and chemical signals to attract and court females^22^ (but see Haniotakis *et al*.^23^ and Baker *et al*.^24^ for an exception in *Bactrocera oleae* (Rossi)). During sexual calling, a volatile pheromone blend is typically released from the rectal gland^25,26^ and dispersed by rapid wing beats^27,28^. Pheromones have been described as a key element of the mating system in many tephritid fruit flies, including *Ceratitis capitata* (Wiedemann)^29–31^, *Anastrepha suspensa* (Loew)^32,33^, *Anastrepha obliqua* (Macquart)^34^, *Anastrepha ludens* (Loew)^35,36^, *Bactrocera dorsalis* Hendel^26^, *Bactrocera cucurbitae* Coquillett^26^, *Rhagoletis cerasi* L.^37^ and *Toxotrypana curvicauda* (Gerstaecker)^38^. Female fruit flies tend to be highly selective and may sample courtship of multiple males before choosing a mate^22,39,40^.

Sexual communication is subject to changes through domestication, and this can have important implications for sterile insect technique (SIT) programs. SIT involves the rearing of large numbers of male insects, which are sterilised and released in the field to mate with and prevent reproduction of females in pest populations^41^. Domestication-related changes in sexual communication can reduce sexual compatibility of released sterile males and wild females, and this in turn can reduce efficacy of SIT programs. For example, domesticated fruit flies may have abbreviated and simplified courtship routines^9,12^ and the diurnal patterns of mating activity may shift so that domesticated and wild flies mate at a slightly different time of the day^2,17^.

Differences between laboratory and wild fruit flies in male volatile emissions have been reported in Mediterranean fruit fly, *C*. *capitata* by Vanickova *et al*.^42^ and in Mexican fruit fly, *A*. *ludens* by Bosa *et al*.^43^. In both cases, males from domesticated laboratory colonies that had developed in artificial larval media were found to release more volatiles than wild males that had developed in fruit collected from the field and also exhibited some differences in blend. While these differences might reflect evolutionary changes in volatiles production or release of volatiles by domesticated flies, they might instead reflect differences in larval diet or developmental conditions (e.g., temperature, moisture). The present study more directly addresses effects of domestication on volatile production in a tephritid fruit fly, the Queensland fruit fly, *Bactrocera tryoni* (Froggatt), by comparing colonies at different stages of domestication that have been reared under uniform conditions.

*Bactrocera tryoni* is highly polyphagous and is the most economically damaging insect pest of horticultural crops in eastern Australia^44–46^. Previous studies have highlighted effects of domestication on genetic diversity^47^, adult nutritional requirements^48^, age of first mating, protein consumption and efficiency in converting dietary intake into reproductive output^13^, activity patterns^49^, calling schedule and propensity for indiscriminate mounting^50^ and tolerance of desiccating conditions^51^. To date, the possibility that domestication affects the amount or composition of volatiles released during sexual advertisement by male *B*. *tryoni* has not been considered.

Sexual activity of *B*. *tryoni* is restricted to a period of around 30 minutes at dusk with copulations continuing in darkness^52–54^. During this period, mature males release a volatile blend containing six aliphatic amides^55^ that is secreted and stored in the rectal gland^25,56^. The rectal gland content is released through the anus, rubbed onto the wings using the hind legs, and is dispersed by rapid wing fanning that produces distinctive audible pulses of buzzing (generally referred to as ‘calling’)^53,56,57^. The volatiles produced by *B*. *tryoni* males rectal glands and actively dispersed during sexual calling have been generally interpreted as a ‘sex pheromone’^53,58–63^. While the function of specific components of the blend remain to be elucidated, virgin females are attracted to groups of calling males^62^ or volatiles from crushed glands^59,63^.

For successful SIT programs the released sterile males must succeed in attracting prospective mates. The release of volatiles during sexual calling is a prominent element of the *B*. *tryoni* mating system, and domestication-related changes in composition or amount of released volatiles could diminish efficacy of SIT programs. Pheromones are commonly important both for species discrimination and mate choice in insects^64,65^. Accordingly, changes that render the released males less likely to be recognised as conspecifics by wild females, or to be recognised but assessed as ‘unattractive’, would impinge on success of SIT. The present study considers whether the amount or composition of volatiles emitted during sexual advertisement by male *B*. *tryoni* changes through domestication.

## Materials and Methods

### Insects

Pupae of *Bactrocera tryoni* were obtained from colonies that were already established from locally collected infested fruit at laboratories in Brisbane (Queensland Department of Agriculture, Fisheries and Forestry), Cairns (Queensland Department of Agriculture, Fisheries and Forestry), and Sydney (New South Wales Department of Primary Industries, Ourimbah). From each locality we obtained two colonies that differed in domestication history: a young colony that had been maintained for five generations or fewer and an old colony that had been maintained for 20 generations or more (Brisbane G5 and G25, Cairns G5 and G25 and Sydney G2 and G20). Approximately 500 pupae of each colony were housed in 47.5 × 47.5 × 47.5 cm fine mesh cages (Megaview Bugdorm 4S4545, Taiwan). Flies were fed sugar and hydrolysate yeast (MP Biomedicals LLC) separately and were provided water *ad libitum* through a soaked sponge. All cages were maintained in a controlled environment room at 25 ± 0.5 °C, 65 ± 5% RH, and a 12:12 LD photoperiod with a simulated dawn and dusk in which lights ramped up or down over 30 minutes, respectively. To avoid variations in volatile production due to variations in rearing conditions or diet preparation at each of the source laboratories^42,66^, each colony was reared through one generation at Macquarie University in the same room using standard carrot diet^67^. Approximately 1400 eggs (0.1 mL of eggs suspended in water) were pipetted onto 300 g of carrot diet in a 500 mL plastic container, which was then covered with a lid. After one week, the lids were removed, and the containers were transferred to 12.5 L plastic boxes that contained a 1 cm deep layer of fine vermiculite. Third instar larvae exited the larval diet and pupated in the vermiculite. Pupae were separated by gently sieving the vermiculite 2–3 days before the expected emergence date. Approximately 500 pupae of each colony were kept in a 47.5 × 47.5 × 47.5 cm fine mesh cage (Megaview Bugdorm 4S4545, Taiwan) for emergence. Male flies were transferred to a 1 L clear plastic cage within three days after emergence and were provided sugar, yeast hydrolysate and water. No calling or mating was observed before separating the flies. Eight cages of 30 males were set up for each of the six colonies (three regions, one old and one young colony from each region) and held until 15–20 days old when they are sexually mature^13,48^ for use in volatile collections.

### Volatile Collection

Groups of 30 virgin males (15–20 days old) were placed in a cylindrical glass chamber (150 mm long × 40 mm ID) 30 minutes before dusk. A charcoal-filtered air stream was passed through the glass chamber at a flow rate of 1 L/min. The volatiles released by males during sexual calling at dusk were collected on 50 mg of Tenax-GR traps fitted with glass wool plugs in a 5 cm long glass tube. Volatile collection lasted 1.5 h and was carried out in the same room conditions under which the flies were maintained. Neither food nor water was provided during volatile collections. Volatiles were eluted from Tenax traps using 1.5 mL of hexane (HPLC grade) and were stored at −20 °C. For GC-MS analysis samples were concentrated to 800 μL under a gentle stream of nitrogen. As an internal standard for quantification an aliquot of tridecane stock solution (1.1 mg mL^−1^) was added to each sample to obtain a final concentration of 5.5 µg mL^−1^. Six to eight replicates were collected and analysed for each of the six colonies (three regions, one old and one young colony from each region). To distinguish between compounds released by the flies and any possible contaminants an air control sample, comprising an empty glass chamber, was run and analysed along with every volatile collection.

Before each use, Tenax traps were thermally conditioned by heating them in a nitrogen stream (75 mL/min) at 200 °C for three hours. Glass chambers were washed with water containing 5% Extran, rinsed with hot tap water, and heated at 200 °C for 18 hours. Activated charcoal filters were conditioned by heating at 200 °C for 18 hours^68^.

### GC-MS Analysis

GC-MS analysis was carried out on a Shimadzu GC 2010 spectrometer equipped with a split/splitless injector, a Restek Rxi-5Ms fused silica capillary column (30 m × 0.25 mm, 0.25 μm film) and a mass spectrometer. Helium was used as a carrier gas with a constant flow of 1 mL/min. The column temperature was initially set at 40 °C and held for 3 minutes, then increased to 250 °C at a rate of 10 °C/min and held at 250 °C for 6 minutes. The mass spectrometer was operated in the electron impact mode at 70 eV. Temperatures of injector and detector were set at 270 and 290 °C, respectively. Data acquisition rate was 20 Hz (scans/s) for the mass range of 35–440 amu. One microlitre of sample was injected in the splitless mode. Data were processed using GCMS Postrun Analysis software. Synthetic samples of the six target amides that have previously been reported as the major components of *B*. *tryoni* male rectal gland contents, *N*-(2-methylbutyl)acetamide, *N*-(3-methylbutyl)acetamide, *N*-(2-methylbutyl)propanamide, *N*-(3-methylbutyl)propanamide, *N*-(2-methylbutyl)isobutyramide and *N*-(3-methylbutyl)isobutyramide^55^, were analysed as authentic samples (see supplementary information for synthesis details). The volatile compounds released by *B*. *tryoni* males were then confirmed by comparing their retention time and mass fragmentation pattern to the synthetic standards.

### Statistical Analysis

Each chromatographic peak was analysed in terms of its area and the area percentage of the specific peak relative to the sum of the areas of all peaks in the chromatogram. To reduce variation across GC-MS runs, compound area was standardized by the internal standard. This was accomplished by multiplying each area by the ratio of the median internal standard of all runs to each run’s internal standard. For several compounds, low concentrations meant that some samples lacked discriminable chromatographic peaks. These values were set to half of the minimum measured area within each experiment. One of the compounds, *N*-(2-methylbutyl)isobutyramide (c5), was excluded from the analysis, because too many samples were below the detection threshold.

#### Experiment 1: Comparing young and old colonies from different regions

Volatiles released by males from young and old colonies from Brisbane, Cairns and Sydney were compared. To test if young and old colonies differed in any of the released compounds, we ran a linear mixed model in which the peak area of each compound was the response variable. The predictors in the model were region (Brisbane, Cairns or Sydney), age of colony (young or old), and the selected compounds (c1–c4, c6). To test if young and old colonies differed in representation of any particular compound within the same region, we included three-way interaction (compound by age by region) as well as all lower interactions. A pairwise comparison between young and old colonies for each compound and region was performed with a Holm’s p-value adjustment for multiple comparisons. As compounds were likely correlated within a replicate and compounds may have different error variances, a random effect for sample runs was included for each compound, and residual error estimates were modeled separately for each compound. To satisfy normality assumptions and to enable comparisons between compounds on different scales, the response variable was log-transformed. All analyses were performed in R^69^ using the *lme*() function in the *nlme* package^70^. Model assumptions were assessed using graphical analyses of the Pearson residuals.

#### Experiment 2: Changes within an aging colony

Using only the two colonies from Brisbane, we investigated the changes that occur in the amount of volatiles released as the number of generations increases, that is, as the young colony ages. After collecting volatiles from the young (5 generations) and old (25 generations) Brisbane colonies in Experiment 1, the volatiles released by males from these colonies were collected two more times: when the young colony of Experiment 1 was 12 and 15 generations and the old colony of Experiment 1 was 35 and 38 generations.

For this analysis, we asked whether volatile emissions changed directionally over the three sampling episodes. Similar to Experiment 1, we ran a linear mixed model in which the peak area of each compound was the response variable. The predictors were colony age (young or old), sampling episode (first [Experiment 1], second or third sampling), and the individual compounds (c1–c4, c6). All two-way and three-way interactions between colony age, sampling episode and compound were included. Using backward model selection, interactions were removed if the log-likelihood ratio test was not significant. As for Experiment 1, a random effect for sample run was included for each compound and residual error estimates were modeled separately for each compound. However, after examination of the residuals, there appeared to be higher variance in the first sampling episode (5 and 25 generations in Experiment 1) compared to later sampling episodes (12 and 15 generations for the young colony, 35 and 38 generations for the old colony). Therefore, subsequent models were compared in which residual error estimates were modeled separately for (1) each sampling episode and (2) both sampling episode and compound. The best model allowed for variation just across the sampling episode of volatile collection. Analyses were performed in R^69^ using the *lme()* function in the *nlme* package^70^. Model assumptions were assessed using graphical analyses of the Pearson residuals.

## Results

### Experiment 1: Comparing young and old colonies from different regions

**Figure 1:**
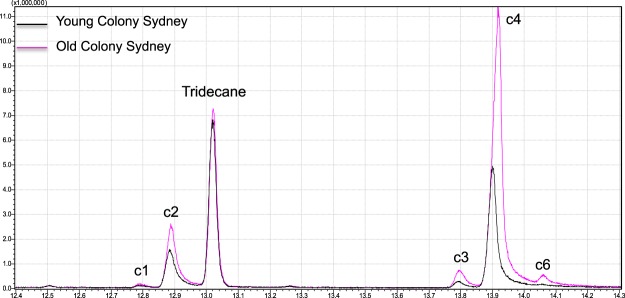
Gas chromatogram comparing volatiles released during sexual calling by *Bactrocera tryoni* males from the young (G2) and old colony (G20) from Sydney. c1: *N*-(2-methylbutyl)acetamide, c2: *N*-(3-methylbutyl)acetamide, c3: *N*-(2-methylbutyl)propanamide, c4: *N*-(3-methylbutyl)propanamide, and c6 *N*-(3-methylbutyl)isobutyramide. Tridecane was used as internal standard.

**Figure 2:**
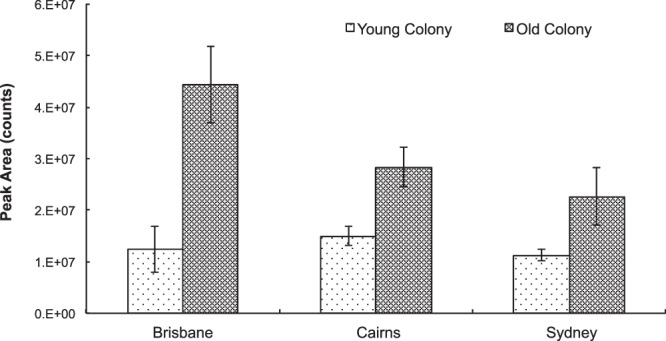
Peak area (Mean ± SE) of volatiles released during sexual calling by *Bactrocera tryoni* males from young and old colonies that were established from three geographical regions, Brisbane, Cairns and Sydney.

**Figure 3:**
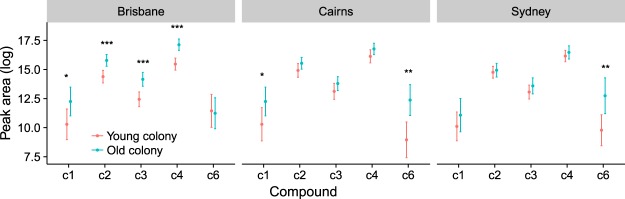
Model estimates for each compound present in the blend released by *Bactrocera tryoni* males from different aged colonies that were established from three geographical regions, Brisbane, Cairns and Sydney. Estimates are shown with 95% CI error bars derived from the statistical model. *P < 0.05, **P < 0.01, ***P < 0.001.

*Bactrocera tryoni* males from all the colonies tested released a blend of six aliphatic amides (c1 to c6), as reported by Bellas & Fletcher^55^. The most abundant compound was *N*-(3-methylbutyl)propanamide, (c4), which comprised ca. 70% of the blend, followed by *N*-(3-methylbutyl)acetamide, (c2) which comprised ca. 20% of the blend. The remaining 10% comprised *N*-(2-methylbutyl)acetamide (c1), *N*-(2-methylbutyl)propanamide (c3), *N*-(2-methylbutyl)isobutyramide (c5) and *N*-(3-methylbutyl)isobutyramide (c6) (Fig. 1). In each of the three regions, males from old colonies released more volatiles overall than males from young colonies (Fig. 2). However, the differences between young and old colonies in the abundance of individual compounds and the magnitude of the increase varied by compound and by region (Fig. 3, Tables 1 and 2). The increase from young to old colony from Brisbane ranged from 0.8 to 7.1 fold with all of the compounds significantly different, except c6. In colonies from Cairns only c1 and c6 were significantly more abundant in the blend of flies from the old colony, with the largest proportional increased in c6 (30.4 fold). In colonies from Sydney only c6 was significantly more abundant in the blend of flies from the old colony (19.3 fold). Interestingly, the compound that differed to the greatest extent between young and old colonies from Cairns and Sydney (c6) was the only one not significantly different between the young and old colony from Brisbane. Each of the five amide compounds tested was more abundant in the volatiles emitted by the old colony from at least one of the three regions (Table 2, Fig. 3).

**Table 1:** Statistical comparisons of the quantity of volatiles released by *Bactrocera tryoni* males.

Variable	F value	DF num	DF dem	*P*
Age	13.83	1	37	<0.001
Compound	2231.72	4	148	<0.001
Region	0.23	2	37	0.80
Age: Compound	3.21	4	148	0.015
Age: Region	7.08	2	37	0.0025
Compound: Region	2.16	8	148	0.034
Age: Compound: Region	4.11	8	148	<0.001

Age indicates colony age (young vs. old), Compound indicates each compound (c1–c4, c6), Region indicates the three geographical regions tested (Brisbane, Cairns and Sydney).

**Table 2:** Statistical comparisons of the quantity of each of the five compounds released by *Bactrocera tryoni* males within colonies from each region.

Region	Compound	Estimate	SE	DF	*P*	*e* ^*x*^
Brisbane	c1	1.96	0.89	37	0.035	7.11
	c2	1.40	0.37	37	<0.001	4.05
	c3	1.72	0.43	37	<0.001	5.56
	c4	1.67	0.35	37	<0.001	5.29
	c6	−0.21	0.96	37	0.83	0.81
Cairns	c1	1.96	0.93	37	0.043	7.10
	c2	0.61	0.38	37	0.12	1.84
	c3	0.68	0.45	37	0.14	1.97
	c4	0.64	0.37	37	0.09	1.90
	c6	3.42	1.00	37	0.0016	30.45
Sydney	c1	0.97	0.93	37	0.31	2.64
	c2	0.18	0.38	37	0.65	1.19
	c3	0.53	0.45	37	0.25	1.70
	c4	0.31	0.37	37	0.40	1.37
	c6	2.96	1.00	37	0.0054	19.35

(*e*^*x*^) represents the fold-increase (or decrease) from young to old colony.

### Experiment 2: Changes within an aging colony

**Figure 4:**
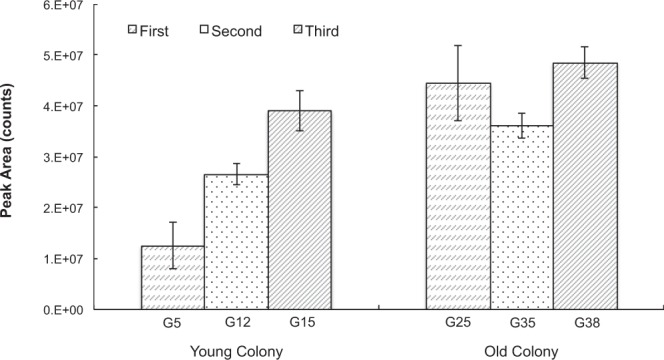
Peak area (Mean ± SE) of volatiles released during sexual calling by *Bactrocera tryoni* males from a young and an old colony from Brisbane, illustrating significant generational changes as total output of the young colony approaches that of the old colony across generations.

**Figure 5:**
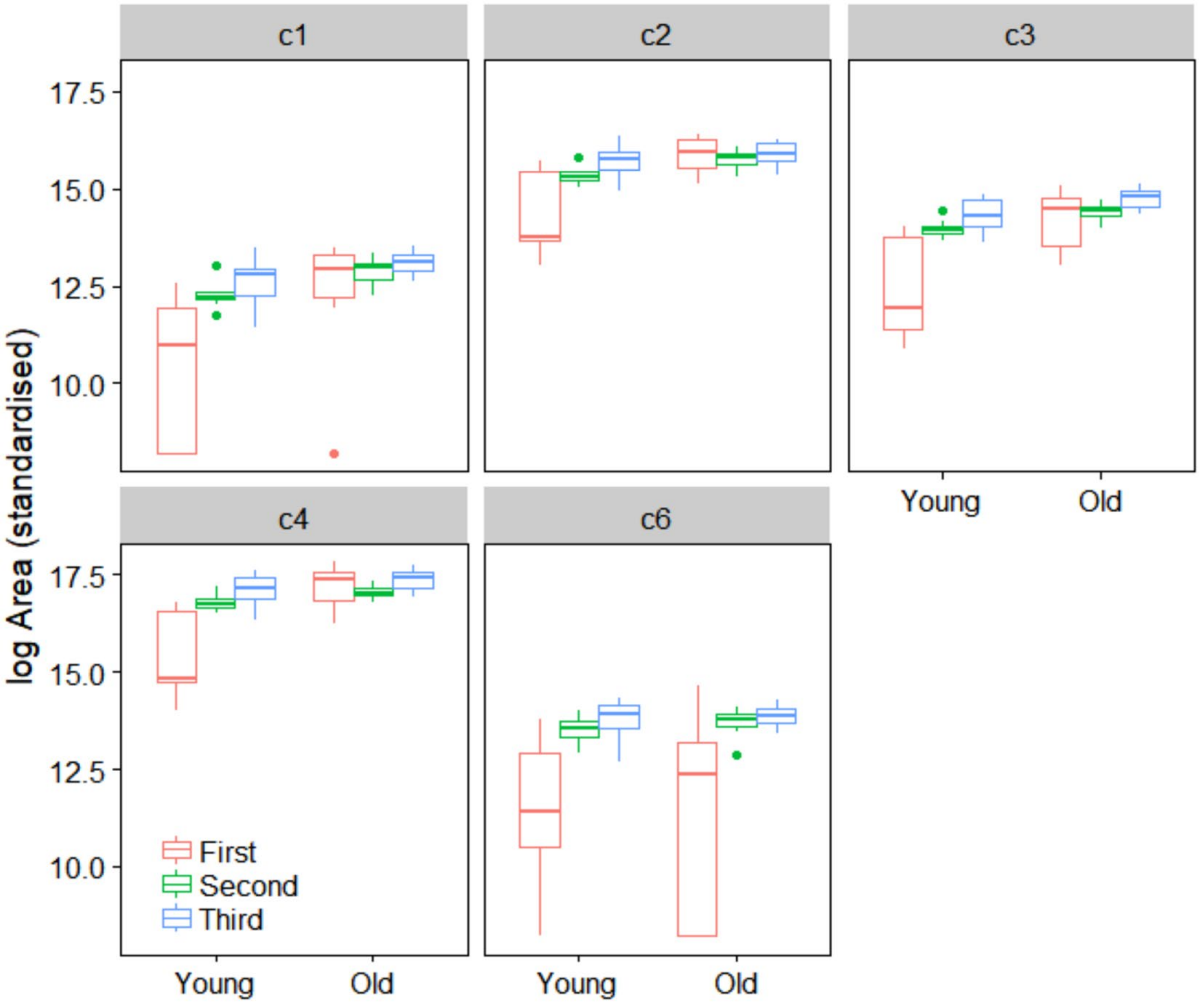
Model estimates for each compound present in volatiles released by *Bactrocera tryoni* males from and a young and an old colony from Brisbane. Estimates are shown with 95% CI error bars derived from the statistical model, illustrating significant generational changes as output of components in emissions of the young colony approaches those of the old colony across generations.

Very similar patterns to those of Experiment 1 were observed in Experiment 2 using only the colonies from Brisbane. Males from the old colony released more volatiles than males from the young colony (Fig. 4), although the difference between the young and old colony decreased over the three sampling episodes. The amount of volatiles released by males from the old colony was 3.3 fold greater than the young colony on the first sampling episode (5 vs. 25 generations) (estimate of difference on log scale = 1.2 ± 0.36, *P* < 0.001) but was only 1.5 fold greater on the second sampling episode (12 vs. 35 generations) (estimate = 0.39 ± 0.13, *P* = 0.004) and 1.4 fold greater on the third sampling episode (15 vs. 38 generations) (estimate = 0.30 ± 0.11, *P* = 0.009). The young colony changed much more than the old colony over the sampled period, with a 2.1 fold increase between generation 5 and generation 12 and an additional 1.5 fold increase between generation 12 and generation 15 (Fig. 5, Table 3). Almost all compounds were more abundant in the older colony, except c6 which did not change over the three sampling episodes and c2 that showed differences only in the second sampling episode (Table 4). The greatest difference between the old and the young colonies was observed in the first sampling episode (Table 4).

**Table 3:** Statistical comparisons of the quantity of volatiles released by *Bactrocera tryoni* males from colonies from Brisbane tested over sequential generations of domestication.

Variable	F value	DF num	DF dem	*P*
Age	15.1	1	214	<0.001
Compound	4332.4	4	214	<0.001
Time	36.9	2	214	<0.001
Age: Compound	4.8	4	214	<0.001
Age: Time	3.1	2	214	0.047
Compound: Time	2.2	8	214	0.032
Age: Compound: Time	0.8	8	214	0.57

Age indicates colony age (old vs. young), compound indicates each compound (c1–c4, c6), and time indicates the episode of volatiles collection (first, second or third).

**Table 4:** Statistical comparisons of quantity of each of the five compounds released by *Bactrocera tryoni* males from young and old colonies from Brisbane tested over sequential generations of domestication.

Compound	Time	Estimate	SE	DF	P	(${e}^{x}$)
c1	1	1.83	0.77	216	0.018	6.24
	2	0.61	0.19	216	0.0018	1.84
	3	0.49	0.16	39	0.0040	1.64
c2	1	1.28	0.76	216	0.09	3.61
	2	0.39	0.16	216	0.015	1.48
	3	0.18	0.14	39	0.19	1.20
c3	1	1.63	0.76	216	0.033	5.10
	2	0.43	0.13	216	<0.001	1.54
	3	0.48	0.11	39	<0.001	1.61
c4	1	1.58	0.76	216	0.039	4.84
	2	0.30	0.12	216	0.012	1.35
	3	0.27	0.11	39	0.017	1.30
c6	1	−0.23	0.77	216	0.77	0.80
	2	0.19	0.18	216	0.28	1.21
	3	0.08	0.15	39	0.59	1.09

Time indicates the episode of volatiles collection (first, second or third), (*e*^*x*^) represents the fold-increase (or decrease) from young to old colony.

## Discussion

The present study reports qualitative and quantitative changes in the volatiles released during sexual advertisement by sexually mature *B*. *tryoni* males as a result of domestication. While there was regional variation in which components differed in abundance between young and old colonies, all comparisons were consistent in showing a substantial increase in amount of volatiles released by males from older colonies.

Volatile emissions are an important element of sexual performance in most tephritid fruit flies, being key for both long and short-range attraction of sexually receptive females and during courtship^22,32,38,61,71,72^. In nature, male fruit flies commonly release volatiles in loose aggregations where attracted females sample and choose amongst males as prospective mates^32,39,73^. In contrast, under laboratory and factory conditions adult flies are held under extremely crowded conditions^2,7^. In a crowded cage filled with volatiles from hundreds, or even tens of thousands, of calling males, it would be difficult for a female to discriminate among the volatiles produced by different males. Under these circumstances, adult males might release more volatiles to overcome a chemically noisy environment when calling for mates and when courting. In some fruit flies, differences in the amount of volatiles released, or even small differences in the proportion of each compound, can be biologically important^42,43,74^. Although synthesis may be physiological costly, flies in laboratory or factory settings usually have constant access to a highly nutritious diet and are released from the costs of many challenges of nature such as foraging, dispersal, predator evasion, and fluctuations in temperature and humidity. Relaxation of these factors that might constrain volatile production in nature could support the evolution of increased capacity for volatile production and calling in laboratory or factory environments.

Production and composition of fruit fly volatiles can be affected by nutrition, mating status, and age^75,76^. For example, sexually mature Caribbean fruit fly, *Anastrepha suspensa* (Loew) males that have no access to protein overnight produce less volatiles the following day^74^. In some studies that varied nutrition through the development phase it is difficult to disentangle the direct effects of nutrition on volatile production and the indirect effects of nutrition caused by group differences in maturation. For example, Nation^77^ found that *A*. *suspensa* that have been deprived of protein since emergence produce much less volatiles than do males that have been provided protein. Liedo *et al*.^66^ found that males of *A*. *ludens* and *Anastrepha obliqua* (Macquart) that were fed only sugar, mango, orange or pear juice produced less volatiles than did males fed yeast hydrolysate and sugar (1:3) since emergence. In these studies, however, because treatment was imposed through the adult development phase and volatiles were then assessed over the same age range for all diet groups irrespective of how those diets affect development rate, it is not possible to ascertain the extent to which the findings reflect differential maturation or differential rectal gland production by mature adults. In the present study, larvae and adults of all the colonies tested were fed the same diet; carrot media for larval development followed by sugar and hydrolysate yeast for adults. Because all the colonies were subjected to the same diets, the differences in emission of young and old colonies cannot be readily explained as an effect of diet. However, while diet provided was consistent amongst the colonies, there might be differences between the young and old colonies in ability to access and use nutrients from yeast. Meats *et al*.^13^ found that over generations of domestication *B*. *tryoni* became more efficient at converting yeast intake into reproductive output. This trend may reflect adaptation to the specific nutrient profile of the yeast hydrolysate and sucrose that flies are typically provided in laboratory environments. Similarly, changes in volatile emissions of male *B*. *tryoni* during domestication might reflect evolutionary changes in ability to utilize nutrition from yeast hydrolysate and sucrose to produce or release volatiles.

Differences in development stage more generally could drive differences in volatile emission of males from young and old colonies. As is commonplace in fruit flies^9–11,15^, accelerated reproductive development of adults is a prominent feature of domestication in *B*. *tryoni*^13^. To ensure that males from all colonies were mature when tested, in this study we used males that were between 15 to 20 days of age. Parallel studies of the Brisbane colonies (Pérez, unpublished data), have found that more than 90% of males from both the young and the old colonies mated at 8 days of age, and so the ages used for testing would have ensured maturity in the present study. The relatively greater volatile emission of males from older colonies cannot be easily explained by colony differences in maturation.

If there is a link between volatile emission and sexual success under field conditions, then the increased emission of domesticated flies may confer valuable competitive advantages when these flies are released in SIT programs. However, elevated volatile emission may also present a risk. If synthesis in the rectal gland is costly, then once outside the nutritionally rich factory environment the flies may struggle to find the resources required to sustain high levels of emission. Because they are adapted to a controlled environment where resources are constantly available, domesticated flies may be deficient in ability to respond to resource deficiencies. In the present study we found that males from old colonies emit more volatiles during sexual advertisement than those from young colonies when provided a standard laboratory diet, but it would be interesting to consider the extent to which males from young and old colonies are able to modulate emission in response to resource availability^74^.

The higher levels of volatile emission by males from older colonies may reflect increased production in the rectal glands, increased calling propensity, or more intense calling. Like many fruit flies^22,27,28,38,77–79^, *B*. *tryoni* rapidly fan their wings while emitting volatiles from the rectal glands^53,57^. Because an audible sound is produced, this is generally referred to as ‘calling’. While the sounds produced by wing fanning might have significance as acoustic signals^12,79,80^ they have also been implicated as important for dispersion of rectal gland contents^28,81^. The higher levels of volatile emission by males from older colonies may arise in part from greater propensity to engage in calling behaviour, or more intense calling behaviour, but the differences in volatile blend of young and old colonies, and the differences in compounds that varied significantly between young and old colonies from the three sampled regions (see Fig. 3, Table 2), almost certainly reflect differences in rectal gland production. While we have found clear effects of domestication on amount and blend of volatiles emitted during sexual advertisement by *B*. *tryoni*, the mechanisms underlying these effects, and their functional significance in terms of sexual success, remain to be elucidated.

## Electronic supplementary material

Supplementary Information
