# Supplementary material for: Domestication modifies the volatile emissions produced by male Queensland fruit flies during sexual advertisement
Source: Sci Rep. 2018 Nov 7;8:16503. doi: 10.1038/s41598-018-34569-3 (PMC6220308; doi:10.1038/s41598-018-34569-3)
Supplement: Supplementary file 1 — Supplementary Information [file 41598_2018_34569_MOESM1_ESM.docx]

**Supplementary Information for**

**Domestication modifies the volatile emissions produced by male Queensland fruit flies during sexual advertisement**

Jeanneth Pérez^1,3^*, Soo Jean Park^2,3^ & Phillip W. Taylor^1,3^

*^1^ Department of Biological Sciences, Macquarie University, Sydney, NSW 2109, Australia.*

*^2^ Department of Molecular Sciences, Macquarie University, Sydney, NSW 2109, Australia.*

*^3^ Australian Research Council Industrial Transformation Training Centre for Fruit Fly Biosecurity Innovation, Macquarie University, Sydney, NSW 2109, Australia.*

Corresponding author:

Jeanneth Pérez, Department of Biological Sciences, Macquarie University, Sydney, NSW 2109, Australia.

E-mail: [jeanneth.perez@mq.edu.au](mailto:jeanneth.perez@mq.edu.au)

# General Procedures

^1^H and ^13^C Nuclear Magnetic Resonance (NMR) spectra were recorded using a Bruker Avance DPX 400 operating at 400 MHz for ^1^H NMR and at 101 MHz for ^13^C NMR. CDCl_3_ was used as a solvent for all NMR samples. ^1^H NMR chemical shifts are reported in parts per million (δ) referenced to the proton signal of the deuterated solvent (CDCl_3_; 7.26 ppm), whereas ^13^C NMR chemical shifts are reported with reference to the carbon signals of the deuterated solvent (CDCl_3_: 77.16 ppm) unless otherwise stated. Mass spectra were recorded on Shimadzu 2010 GCMS spectrometer. Ionization of samples was carried out using electron impact (EI). All reagents were purchased from Sigma-Aldrich, Merck, Ajax Finechem or Alfa-Aesar and used without further purification.

# Synthesis of the amides

Amide synthesis was completed by the reaction of an amine with an acid anhydride in water, modified from a previously reported method^1^. In brief, to a solution of an amine (50 – 60 mmol scale, 1 eq) in water (50 mL) was added acid anhydride (1.5 eq). The reaction mixture was stirred at room temperature for 0.5 – 2 h. After the completion of a reaction monitored by GC, the aqueous reaction mixture was extracted with ethyl acetate (50 mL × 3). The combined organic layer was, washed with 5% NaHCO_3_ solution (150 mL), dried over Na_2_SO_4_ and concentrated under reduced pressure to give the product, which was purified by vacuum distillation.

*N*-(2-Methylbutyl)acetamide (c1). 0.5 h, clear liquid, 5.55 g obtained (50 mmol scale, 86% yield). ^1^H NMR (400 MHz, CDCl_3_) δ 0.83 (6 H, m, CHCH_2_C**H**_3_ and CH_2_C**H**_3_), 1.07 (1 H, apparent sep, *J* = 6.6, C**H**CH_3_), 1.29 – 1.53 (2 H, m, C**H**_2_CH_3_), 1.93 (3 H, s, C**H**_3_CO), 2.94 – 3.13 (2 H, m, NC**H**_2_), 6.33 (1 H, bs, N**H**); ^13^C NMR (101 MHz, CDCl_3_) δ 11.2, 17.1, 23.1, 27.0, 34.8, 45.4, 170.6; GCMS (EI) *m/z* (%) 129 (M^+^, 8), 100 (M^+^−CH_2_CH_3_, 38), 72 (M^+^−CHCH(CH_3_)CH_2_CH_3_, 100). This compound is known, but spectral data are not available in the literature.

*N*-(3-Methylbutyl)acetamide (c2). 0.5 h, clear liquid, 5.36 g obtained (57 mmol scale, 73% yield). ^1^H NMR (400 MHz, CDCl_3_) δ 0.85 (6 H, d, *J* = 6.6, CH(C**H**_3_)_2_), 1.33 (2 H, m, C**H**_2_CH_3_), 1.56 (1 H, sep, *J* = 6.7, C**H**), 1.92 (3 H, s, C**H**_3_CO), 3.18 (2 H, m, NC**H**_2_), 6.21 (1 H, bs, N**H**); ^13^C NMR (101 MHz, CDCl_3_) δ 22.4, 23.1, 25.8, 38.0, 38.3, 170.4; GCMS (EI) *m/z* (%) 129 (M^+^, 5), 114 (M^+^−CH_3_, 12), 73 (M^+^−CH_2_CH_2_CH(CH_3_)_2_, 100). MS data match with those in the literature^2^. NMR data are not available in the literature.

*N*-(2-Methylbutyl)propanamide (c3). 1 h, clear liquid, 4.50 g obtained (50 mmol scale, 63% yield); ^1^H NMR (400 MHz, CDCl_3_) δ 0.82 (6 H, m, overlapped CH(CH_3_)CH_2_C**H**_3_ and COCH_2_C**H**_3_), 1.06 (4 H, m, overlapped C**H**C**H**_3_), 1.41 (2 H, m, CHC**H**_2_CH_3_), 2.15 (2 H, q, *J* = 7.6, CH_3_C**H**_2_CO), 3.04 (2 H, m, HNC**H**_2_), 6.06 (1 H, bs, NH); ^13^C NMR (101 MHz, CDCl_3_) δ 10.1, 11.2, 17.1, 27.0, 29.7, 34.9, 45.1, 174.1; GCMS (EI) *m/z* (%) 143 (M^+^, 10), 86 (·CH_3_CH_2_CH(CH_3_)CH_2_NH ^+^, 75), 57 (CH_3_CH_2_CHCH_3_^+^, 100). This compound is known, but spectral data are not available in the literature.

*N*-(3-Methylbutyl)propanamide (c4). 2 h, clear liquid, 5.75 g obtained (51 mmol scale, 79% yield); ^1^H NMR (400 MHz, CDCl_3_) δ 0.80 (6 H, m, CH(C**H**_3_)_2_), 1.04 (3 H, t, *J* = 7.6, CH_2_C**H**_3_), 1.29 (2 H, m, C**H**_2_ CH), 1.51 (1 H, sep, *J* = 6.7, C**H**), 2.11 (2 H, q, *J* = 7.6, C**H**_2_CH_3_), 3.15 (2 H, m, HNC**H**_2_), 6.38 (1 H, bs, N**H**); ^13^C NMR (101 MHz, CDCl_3_) δ 10.0 ; 22.9, 25.2, 29.7, 36.7, 37.5, 174.6; GCMS (EI) *m/z* (%) 143 (M^+^, 8), 128 (M^+^−CH_3_, 10), 114 (M^+^−CH_2_CH_3_, 9) 57 ((CH_3_)_2_CHCH_2_^+^ , 100). This compound is known, but spectral data are not available in the literature.

*N*-(2-methylbutyl)isobutyramide (c5). 2 h, colourless needles, 6.83 g obtained (50 mmol scale, 87% yield). ^1^H NMR (400 MHz, CDCl_3_) δ 0.81 (6 H, d, *J* = 6.6, COCH(C**H**_3_)_2_), 1.04 (6 H, d, *J* = 6.9, CH(C**H**_3_)_2_), 1.30 (2 H, m, C**H**_2_ CH), 1.52 (1 H, sep, *J* = 6.9, C**H**(CH_3_)_2_), 2.31 (1 H, sep, *J* = 6.6, C**H**CO), 3.27 (2 H, m, HNC**H**_2_), 6.21 (1 H, bs, N**H**); ^13^C NMR (100 MHz, CDCl_3_) δ 19.7, 22.9, 25.2, 35.4, 36.7, 37.8, 177.0; GCMS (EI) *m/z* (%) 157 (M^+^, 8), 142 (M^+^−CH_3_, 12), 114 (M^+^−CH(CH_3_)_2_, 16), 101 (M^+^−CH_3_ and CH(CH_3_)_2_, 50), 71 (·CH_3_CH_2_CON^+^, 100). This compound is known, but spectral data are not available in the literature.

*N*-(3-methylbutyl)isobutyramide (c6). 1 h, 4.99 g obtained (60 mmol scale, 53% yield). ^1^H NMR (400 MHz, CDCl_3_) δ 0.90 (6 H, m, *J* = 6.6, CHCH_2_C**H**_3_ and CH_2_CH(C**H**_3_)CH_2_), 1.13 (1 H, m, C**H**(CH_3_)CH_2_) 1.16 (6 H, d, *J* = 6.9, CH(C**H**_3_)_2_), 2.37 (1 H, sep, *J* = 6.9, C**H**(CH_3_)_2_), 3.12 (2 H, m, HNC**H**_2_), 5.65 (1 H, bs, N**H**); ^13^C NMR (100 MHz, CDCl_3_) δ 11.4, 17.2, 19.8, 27.1, 35.0, 35.9, 45.0, 177.2; GCMS (EI) *m/z* (%) 157 (M^+^, 10), 114 (M^+^− (CH_3_ and CH_2_CH_3_), 45), 43 ((CH_3_)_2_CH^+^, 100). This compound is known, but spectral data are not available in the literature.

# **References**

1. Naik, S., Bhattacharjya, G., Talukdar, B. & Patel, B.K. Chemoselective acylation of amines in aqueous media. *Eur. J. Org. Chem.* **6,** 1254-1260 (2004).

2. Wee, S.L. & Tan, K.H. Female sexual response to male rectal volatile constituents in the fruit fly, *Bactrocera carambolae* (Diptera: Tephritidae). *Appl. Entomol. Zool.* **40,** 365-372 (2005).
